# Glioma-associated microglia/macrophages augment tumorigenicity in canine astrocytoma, a naturally occurring model of human glioma

**DOI:** 10.1093/noajnl/vdab062

**Published:** 2021-05-04

**Authors:** Ryan Toedebusch, Ana Cristina Grodzki, Peter J Dickinson, Kevin Woolard, Nicole Vinson, Beverly Sturges, John Snyder, Chai-Fei Li, Ori Nagasaka, Blaire Consales, Karen Vernau, Marguerite Knipe, Vishal Murthy, Pamela J Lein, Christine M Toedebusch

**Affiliations:** 1 Department of Surgical and Radiological Sciences, School of Veterinary Medicine, University of California, Davis, Davis, California, USA; 2 Department of Molecular Biosciences, School of Veterinary Medicine, University of California, Davis, Davis, California, USA; 3 Department of Pathology, Microbiology, and Immunology, School of Veterinary Medicine, University of California, Davis, Davis, California, USA; 4 Riemann Computing, LLC, St. Louis, Missouri, USA

**Keywords:** canine, glioma, microglia, transforming growth factor beta 1

## Abstract

**Background:**

Glioma-associated microglia/macrophages (GAMs) markedly influence glioma progression. Under the influence of transforming growth factor beta (TGFB), GAMs are polarized toward a tumor-supportive phenotype. However, neither therapeutic targeting of GAM recruitment nor TGFB signaling demonstrated efficacy in glioma patients despite efficacy in preclinical models, underscoring the need for a comprehensive understanding of the TGFB/GAM axis. Spontaneously occurring canine gliomas share many features with human glioma and provide a complementary translational animal model for further study. Given the importance of GAM and TGFB in human glioma, the aims of this study were to further define the GAM-associated molecular profile and the relevance of TGFB signaling in canine glioma that may serve as the basis for future translational studies.

**Methods:**

GAM morphometry, levels of GAM-associated molecules, and the canonical TGFB signaling axis were compared in archived samples of canine astrocytomas versus normal canine brain. Furthermore, the effect of TGFB on the malignant phenotype of canine astrocytoma cells was evaluated.

**Results:**

GAMs diffusely infiltrated canine astrocytomas. GAM density was increased in high-grade tumors that correlated with a pro-tumorigenic molecular signature and upregulation of the canonical TGFB signaling axis. Moreover, TGFB1 enhanced the migration of canine astrocytoma cells in vitro.

**Conclusions:**

Canine astrocytomas share a similar GAM-associated immune landscape with human adult glioma. Our data also support a contributing role for TGFB1 signaling in the malignant phenotype of canine astrocytoma. These data further support naturally occurring canine glioma as a valid model for the investigation of GAM-associated therapeutic strategies for human malignant glioma.

**Key Points**

- GAM density increases with increasing tumor grade in canine astrocytoma.
- TGFB1 signaling directly contributes to the malignant phenotype of canine astrocytoma.

**Importance of the Study**

Despite the completion of greater than 450 clinical trials by 2020, malignant glioma remains uniformly fatal. Therapeutic efficacy in standard preclinical models has failed to translate to human patients, highlighting the limitations in accurate prediction of therapeutic response in heterogeneous human glioma. Spontaneous canine glioma may provide a more faithful translational model for human glioma, given their tumor heterogeneity and a naturally developing tumor-immune environment. More specifically, we demonstrate that the glioma-associated microglia/macrophage signature in canine astrocytomas shares strong similarities with that in human adult malignant gliomas. Canine astrocytomas exhibit upregulation of analogous pro-tumorigenic molecules, including upregulation of the canonical TGFB1 signaling axis and associated effects on glioma cell migration and invasion. These data further support the companion canine as a potentially valuable preclinical translational model for human glioma, allowing for the additional, more stringent assessment of promising novel therapeutics showing efficacy in standard rodent model systems.

Malignant gliomas are the most common and aggressive primary brain tumor of adults, with a 5-year survival rate of approximately 5%.^1^ Although immunotherapy has advanced the treatment of non-central nervous system (CNS) tumors, it has, to date, failed to overcome the substantial barrier of immune resistance in the glioma microenvironment. Gliomas have a low tumor mutational burden,^2^ allowing them to evade an anti-tumor immune response. Moreover, as the most abundant glioma-invading cells,^3^ glioma-associated microglia and blood-derived macrophages (GAMs) substantially contribute to the immunosuppressive glioma microenvironment.^4^ In fact, GAM infiltration is positively correlated with glioma grade^5^ and invasiveness,^6^ highlighting their critical role in tumor progression.

While the glioma-microglia signaling axis is complex, transforming growth factor beta 1 (TGFB1) has been identified as a key mediator of glioma progression. Under the influence of glioma-derived TGFB1, GAMs demonstrate negligible anti-tumor activity and are polarized to produce pro-tumorigenic molecules.^7^ Moreover, through a positive feedback paracrine loop,^8^ GAM-derived TGFB1 promotes glioma growth^9^ and invasion.^10^ Therapeutic targeting of TGFB signaling^11^ and GAM recruitment^12^ has demonstrated survival benefits in preclinical rodent models, but these interventions have not been successful in glioma patients. These failed translations may be, in part, due to the incomplete recapitulation of biological tumor heterogeneity and environmental complexity of human patients by orthotopic rodent models. However, it is also possible that TGFB has both pro-tumor and antitumor activities in GBM as in other non-CNS cancers.^13,14^ Therefore, it is critical to develop a comprehensive understanding of the TGFB/GAM axis in glioma to develop refined, specific targets.

Naturally occurring canine gliomas, with biological and environmental complexity, provide a translationally relevant animal model of human glioma. With a comparable incidence,^15,16^ canine gliomas share clinical,^17^ imaging^18^ histopathologic,^19^ and molecular^20,21^ features with human glioma. Limited observations suggest the immune landscape is similar to adult high-grade human glioma, as canine high-grade gliomas exhibit a robust microglia/macrophage infiltrate.^21^ However, the GAM-associated molecular profile and the relevance of the TGFB signaling axis remain critical knowledge gaps in canine glioma biology. Given the relevance of GAM and TGFB in human glioma, our study aims to define the GAM response in canine astrocytoma and determine the relevance of the TGFB signaling axis on the malignant phenotype of canine astrocytoma cells.

## Methods

### Sample Collection

Tissue samples from canine astrocytoma patients were obtained from clinical cases presented to the University of California, Davis Veterinary Medical Teaching Hospital between 2002 and 2018. Case information is provided in Supplementary Table 1. Samples were collected via surgical or image-guided biopsy or necropsy within 1 h following euthanasia. Normal canine brain tissue was collected at necropsy within 1 h following euthanasia from client-owned dogs donated for research with informed consent. Tissue samples were snap-frozen in liquid nitrogen or immersion fixed in 10% neutral buffered formalin and paraffin-embedded. All tumors were histologically classified by a board-certified veterinary pathologist according to the National Cancer Institute-led multidisciplinary Comparative Brain Tumor Consortium^19^ and further subdivided according to the 2016 WHO classification of human tumors of the CNS.^22^

### Immunofluorescence and Confocal Microscopy

Formalin-fixed paraffin-embedded low- (II) and high-grade (III, IV) astrocytomas, as well as normal canine brain, were sectioned at 10 µm and mounted on poly-l-lysine-treated microscope slides. Two sections of the dog were randomly selected from 10 sections for immunostaining within each condition; serial sections were compared across conditions (ie, TGFB1 and Iba-1 proximity). Following deparaffinization, antigen retrieval, and permeabilization (Supplementary Methods), tissue was blocked using normal goat serum (5%) and bovine serum albumin (1%) in Tris-buffered saline (TBS) for 24 h at 4°C. After blocking, tissue was incubated in primary antibody solution for 24 h at 4°C, washed with TBS with 0.5% Triton X-100, and incubated in secondary antibody solution for 1 h at room temperature. See Supplementary Methods for details on antibodies used. Fluoroshield with DAPI (Millipore Sigma) mounting media was used to stain nuclei and coverslips were applied. The entire tumor volume, or similar tissue volume in nontumor samples, was imaged via Leica TCS SP8 STED 3× confocal microscope.

### Image Analysis

Using a random number generator, 20 images per acquisition were selected for analysis of microglia density and morphology. See Supplementary Methods and Supplementary Figure 1A–G for a detailed description of the analysis built using Custom Module Editor for MetaXpress High-Content Imaging System software (Molecular Devices).

### Quantitative RT-PCR

Targeted gene mRNA expression was assessed in high-grade (III, IV) astrocytomas compared to normal cortex. Total RNA isolation and cDNA synthesis were performed as described in Supplementary Methods. Targets included *CCL2*, *LGALS3*, *IL-1B*, *IL-6*, *IL-10*, *SMAD2*, *SMAD4*, *TGFB1*, *TGFBR1*, *TGFBR2*, and *VEGFA*. Primer sets (Supplementary Table 2) were designed using NCBI primer design (https://www.ncbi.nlm.nih.gov/tools/primer-blast/index.cgi). Primer validation and qPCR reactions were carried out as described in Supplementary Methods. Fold difference in expression between normal brain and tumor samples was calculated and plotted using the double delta Ct analysis with β-actin as the control gene for normalization and relative expression.^23^

### Western Blot Analysis

Protein extraction, separation, and immunoblotting of low- and high-grade astrocytoma tissue, as well as normal canine cortex, were performed as previously described.^24^ Western blot targets included TGFB1 (13kD), TGFB1 (44kD) TGFBR1, TGFBR2, SMAD2/3, pSMAD2/3, and Galectin-3. All western blots are representative of 3 independent experiments. See Supplementary Methods for details on antibodies used. Chemiluminescence images were acquired using ChemiDoc XRS+ System (Bio-Rad) after ECL reagent incubation (Thermo Fisher Scientific). Densitometry was performed using ImageLab software v6.0 (Bio-Rad).

### Canine Protein Arrays

Commercially available protein arrays with preselected, canine-validated targets were utilized for an unbiased evaluation of the molecular milieu in high-grade canine astrocytomas compared to normal canine cortex. Canine tissue was prepared as previously described^25^ and analyzed by RayBiotech Life (Peachtree Corners) with standard quality control. In brief, Quantibody Canine Cytokine Arrays (QAC-CYT-1, QAC-CYT-2, QAC-CYT-4) utilized 2 nonoverlapping arrays of antibody pairs to quantify selected molecules. RayBiotech has confirmed no cross-reactivity between antibody pairs and standard controls. See Supplementary Table 3 for the target protein list.

### Cell Lines

We utilized 2 patient-derived canine grade IV astrocytoma cell lines, 1110 and 0514. Genomic integrity was verified by comparison of copy number alterations with parental tumor DNA using Illumina CanineHD SNP array. Cells were verified to express neural progenitor cell markers similar to human astrocytoma cell lines^26^ (SOX2, OLIG2, glial fibrillary acidic protein [GFAP], NES [nestin]) and sequenced to confirm canine origin. Both cell lines were routinely tested mycoplasma free by PCR. Culture conditions are described in Supplementary Methods.

### Cell Proliferation Assay

Cell proliferation was assessed using the CellTiter-Glo luminescent cell viability assay as described in Supplementary Methods.

### Cell Migration and Invasion Assays

Cell migration and invasion following TGFB treatment were assessed by Boyden Chamber assays as described in Supplementary Methods. Migration experiments were repeated following pretreatment with TGFBR1 inhibition. The percentage of cell invasion was calculated as follows: (mean of invading cells/condition divided by the mean of migrating cells/condition) × 100.

### Statistical Analysis

Statistical analysis was performed with GraphPad Prism v8.4.3 and R v3.6.3 software. Data are presented as the mean ± SEM. Cell culture experiments were performed in technical replicates, with 3 biological replicates. Data were tested for normality via Shapiro–Wilks test. An unpaired 2-tailed Student’s *t*-test or analysis of variance with Tukey’s multiple comparisons test was conducted for most comparisons. When cytokines assayed via cytokine array were below the limit of detection in normal canine brain, a linear mixed model was used to examine how a transformed version of the amount of each protein differed between these groups. A random intercept was used to group the observations from each animal together, accounting for any potential correlation in these measurements. To remedy violations in statistical assumptions, the inverse hyperbolic sine transformation was used. The model was evaluated using the “lmer” function from the “lme4” package available for R (version 3.6.3). Contrasts were computed using the emmeans package in R using the function of the same name.

## Results

### Morphologically Altered GAM Diffusely Infiltrated Canine Astrocytoma

**Figure 1.**
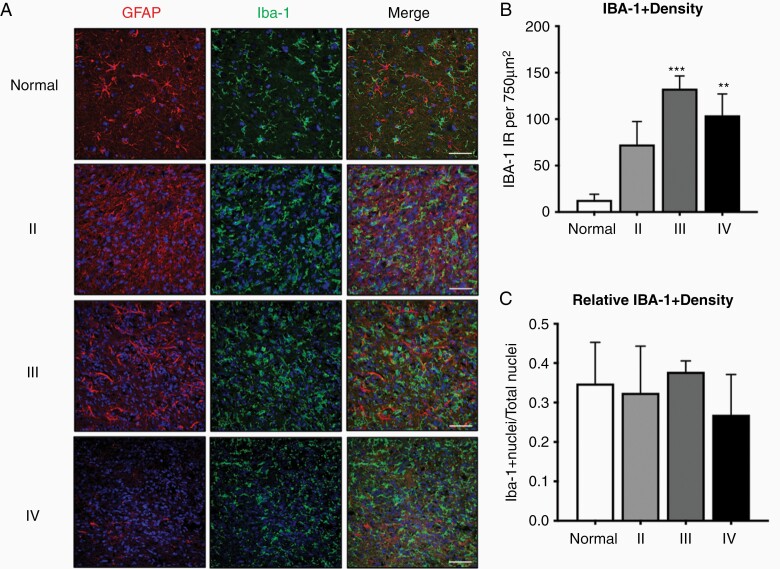
Morphologically altered glioma-associated microglia/macrophages (GAMs) diffusely infiltrated canine astrocytomas. (A) Representative immunofluorescence staining of normal cortex, grade II, III, and IV astrocytomas. Astrocytes, identified by positive immunoreactivity (IR) to glial fibrillary acidic protein (red; GFAP), and microglia, identified by positive IR to ionized calcium-binding adapter molecule 1 (green; Iba-1), were observed among scattered cellular nuclei (blue; DAPI) in normal brain. Thickened, irregular filaments with GFAP IR consistent with neoplastic astrocytes were observed in grade II and III astrocytomas, while a paucity of GFAP IR was noted periodically in grade IV astrocytomas. Diffuse infiltration of cells with Iba-1 IR consistent with microglia/macrophages was noted in all grades of astrocytoma. Scale bars, 20 μm. (B) Grade III (*n* = 3) and IV (*n* = 4) astrocytomas had increased intratumoral Iba-1 IR density compared to normal brain (*n* = 4). (C) The ratio of Iba-1+ nuclei to total nuclei was similar across all groups. Comparisons based on on one-way analysis of variance with post hoc Tukey’s multiple comparisons test. Bars represent group mean with standard error of the mean. **P < .01; ***P < .001.

We analyzed images of paraffin-embedded canine astrocytoma tumor samples (grades II–IV) and normal canine cortex utilizing the microglia/macrophage marker, ionized calcium-binding adapter molecule (Iba-1) and astrocyte marker, GFAP. We observed diffuse Iba-1 immunoreactivity (IR) in all tumor samples (Figure 1A). GAM quantification, as determined by Iba-1+ IR per image, revealed increased GAM density in grade III (133 ± 14; *P* < .001) and grade IV (104 ± 23; *P* < .01) astrocytomas compared to normal cortex (13 ± 6.1; Figure 1B). While GAM density was relatively homogeneous within individual tumors, occasional focal areas of increased GAM density were observed in high-grade (III, IV) astrocytomas. Despite an increase in Iba-1+ IR with progressive tumor grades, the relative Iba-1+ nuclear density remained constant (Figure 1C), representing approximately 30% of all cells within canine glioma across tumor grades. Thus, GAMs diffusely infiltrate canine astrocytomas at a similar density to human glioma.

**Figure 2.**
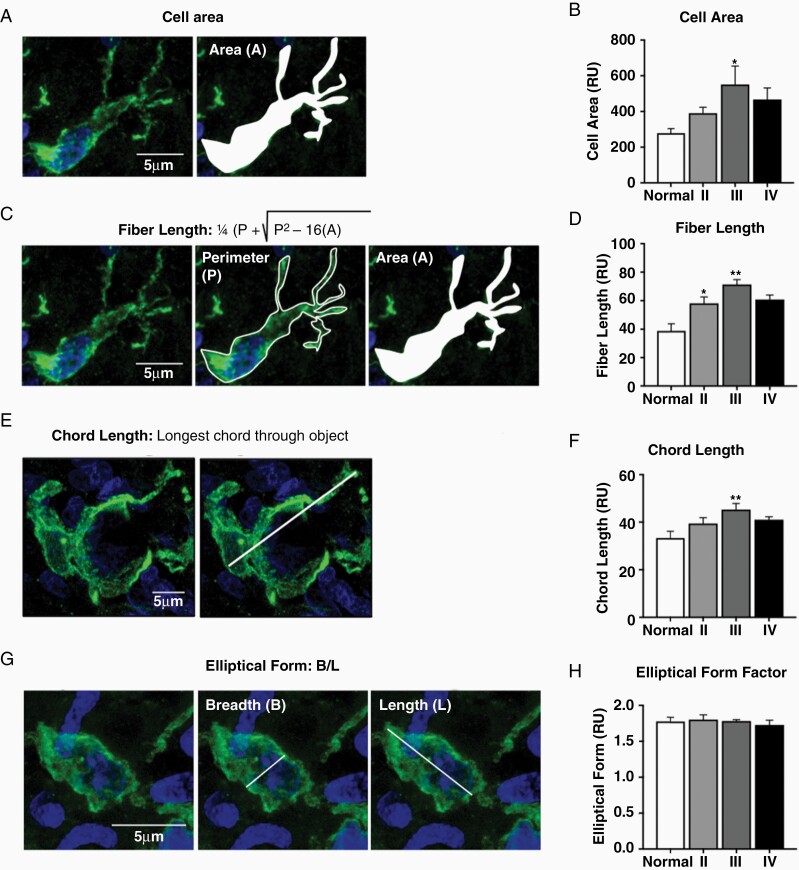
Canine GAMs were a larger, more elongate cellular population. (A) Total cellular area of Iba-1+ cells was measured across all groups. (B) Grade III astrocytoma GAM (*n* = 3) cell area was increased compared to microglia from normal cortex (*n* = 3); *P < .05. (C) Fiber length of all Iba-1+ cells was calculated utilizing cell perimeter and cell area indices. (D) GAM fiber length was increased in all tumor grades (grade II, *n* = 3; grade III, *n* = 3; grade IV, *n* = 4) compared to normal brain (*n* = 3); *P < .05; **P < .01; ***P < .001. (E) Chord length of Iba-1+ cells was determined by measuring the longest straight chord through the entire cell. (F) GAM chord length was increased in grade III astrocytomas (*n* = 3) compared to normal cortical microglia (*n* = 3); **P < .01. (G) Elliptical form factor was calculated by evaluating the ratio of the Iba-1+ cell’s breadth to its length. (H) GAM elliptical form factor averaged 1.75 across all groups; *P* = .8399. Comparisons based on one-way analysis of variance with post hoc Tukey’s multiple comparisons test; *P < .05; **P < .01; ***P < .001. Bars represent group mean with standard error of the mean.

Morphometric analysis of intratumoral Iba-1+ cells revealed that normal canine brain demonstrated the homogeneous distribution of Iba-1+ cells with a small cell body and several branched processes, consistent with a ramified morphology (Figure 2A).^27^ Conversely, GAM morphology across tumor grades reflected a larger cellular population lacking the typical ramified morphology (Figure 2). Compared to normal cortical microglia, the total GAM area was increased in grade III astrocytomas (551 ± 103 vs 279 ± 25; *P* < .05; Figure 2A and B). Additionally, GAM fiber length was increased in grade II (58 ± 4; *P* < .01), grade III (71 ± 3; *P* < .001), and grade IV astrocytomas (61 ± 3; *P* < .01) compared to normal cortical microglia (39 ± 5; Figure 2C and D). Moreover, the average length of the longest chord through a single GAM was increased in grade III astrocytomas (45 ± 3; *P* < .01) compared to normal cortical microglia (33 ± 3; Figure 2E and F). Amoeboid GAMs, characterized by breadth to length ratio of 1, were observed in high-grade astrocytomas (III, IV). However, this was not a predominant morphology, highlighted by the lack of difference in elliptical form factor across tumor grades (*P* = .8399; Figure 2G and H) and normal canine cortical microglia.

### GAM Density and Morphology Were Not Altered in Nontumor Brain

**Figure 3.**
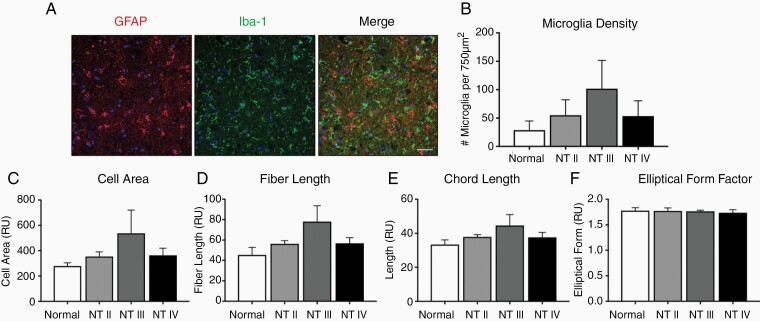
Microglial density and morphology were not different in nontumor-bearing cortex of dogs with astrocytomas. (A) Representative immunofluorescence staining of contralateral, nontumor cerebral cortex of a grade III astrocytoma. Astrocytes, identified by positive IR to glial fibrillary acidic protein (red; GFAP), and microglia, identified by positive IR to ionized calcium-binding adapter molecule 1 (green; Iba-1), were observed among scattered cellular nuclei (blue; DAPI). Scale bars, 20 μm. (B) Microglial density (*P* = .4358), (C) cell area (*P* = .2648), (D) fiber length (*P* = .1256), (E) chord length (*P* = .2505), and (F) elliptical form factor (*P* = .9466) were not different in nontumor cortex of dogs with grade II (*n* = 3), grade III (*n* = 3), or grade IV (*n* = 4) astrocytomas compared to cortex from dogs without tumors. Comparisons based on one-way analysis of variance with post hoc Tukey’s multiple comparisons test. Bars represent group mean with standard error of the mean.

Contralateral normal cortex from astrocytoma-bearing dogs demonstrated a homogeneous distribution of Iba-1+ cells with long, branched processes, interspersed between a homogeneous population of GFAP+ cells (Figure 3A). Systematic analysis across all grades of canine astrocytoma demonstrated no difference in microglia density between the contralateral, tumor-free cortex of dogs affected with astrocytoma compared to normal canine cortex (Figure 3B; *P* = .9914). Similarly, morphometric analysis revealed that microglia from the contralateral, tumor-free cortex had similar cell area (*P* = .2648), fiber length (*P* = .1256), chord length (*P* = .2505), and elliptical form factor (*P* = 0.9466) compared to normal cortical microglia Figure 3C–E.

### Increased GAM Density Is Correlated With a Pro-tumorigenic Molecular Signature in High-Grade Canine Astrocytoma

**Figure 4.**
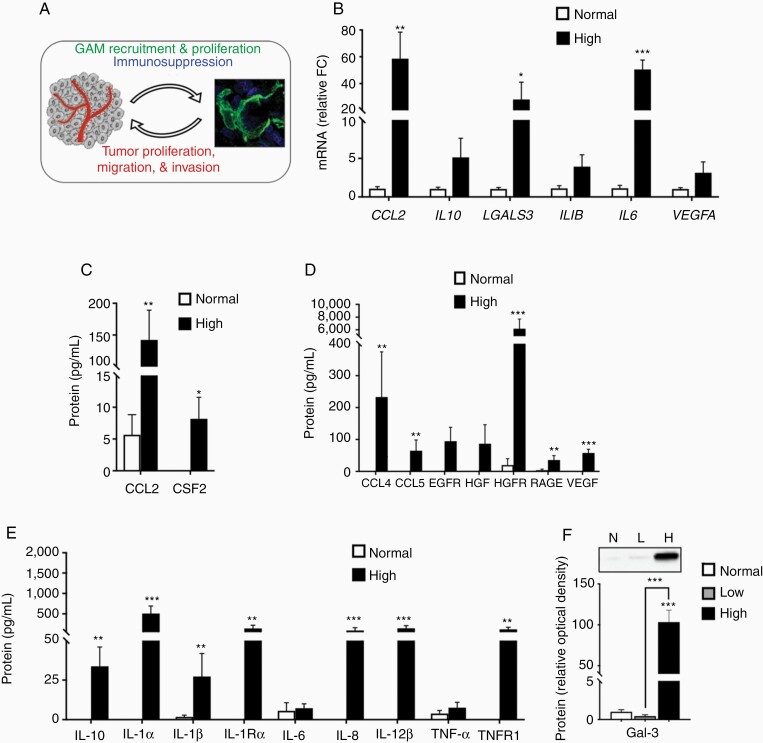
Active recruitment of GAMs was correlated with a pro-tumorigenic molecular signature in high-grade canine astrocytoma. (A) Schematic representation of glioma-GAM crosstalk within the tumor microenvironment promoting tumorigenicity. (B) mRNA levels of GAM chemoattractant and pro-tumorigenic molecules were variably increased in high-grade astrocytoma homogenates (*n* = 4) compared to normal cortex (*n* = 3). Comparisons based on unpaired *t*-test. *P < .05; **P < .01; ***P < .001. (C) Protein levels of CCL2 and CSF2, key mediators of GAM proliferation and recruitment, were increased in high-grade tumor homogenate (*n* = 4) compared to normal cortex (*n* = 3). Comparisons based on unpaired *t*-test; *P < .05; **P < .01. While protein levels of several GAM-derived (D) growth factors and (E) cytokines were undetectable, or present in low concentrations in normal canine cortex (*n* = 3), a robust increase was observed in high-grade tumor homogenate (*n* = 4). Comparisons based on linear mixed model; **P < .01; ***P < .001. (F) Representative immunoblot for galectin-3 (Gal-3), demonstrating a robust increase in Gal-3 IR. Relative optical density of Gal-3 expression was increased 100-fold in high-grade astrocytomas (*n* = 4), compared to normal cortex (*n* = 3) and low-grade astrocytoma (*n* = 3). Comparisons based on one-way analysis of variance with post hoc Tukey’s multiple comparisons test; ***P < .001. Bars represent group mean with standard error of the mean.

In association with increased GAM density, several molecules known for GAM recruitment were elevated in canine astrocytomas. *CCL2* mRNA was increased 59-fold in canine high-grade astrocytoma tumor homogenates compared to normal canine brain (Figure 4B; **P < .01). Consistent with increased mRNA levels, CCL2 protein concentration was increased in high-grade astrocytoma (142.2 ± 47 pg/mL) compared to normal brain (5.7 ± 3 pg/mL; **P < .01; Figure 4C). Additionally, protein levels of colony-stimulating factor 2, while not detectable in normal brain, were abundant in high-grade astrocytoma (8.2 ± 3; **P < .01; Figure 4C).

In addition, we assayed several pro-tumorigenic molecules known to be produced by GAM in human and rodent models of glioma. While below the detectable limit in normal cortex, chemokine C–C ligand 5 (CCL5) and vascular endothelial growth factor (VEGF) concentrations were increased in high-grade astrocytoma (64.8 ± 34 pg/mL; **P < .01; 57.7 ± 12 pg/mL; **P < .001; Figure 4D). Moreover, hepatocyte growth factor receptor (HGFR) was the most highly expressed molecule in this high-grade canine astrocytoma cohort (6172 ± 1533 vs 20 ± 19.9 pg/mL in normal cortex; ***P < .001; Figure 4D). While we did not detect differences in mRNA levels of interleukin-10 (*IL-10*), protein levels were highly abundant in high-grade astrocytoma (33.7 ± 12 pg/mL; **P < .01; Figure 4B and E). Additionally, several cytokines, including the IL-1 family, were increased in high-grade astrocytoma (Figure 4E). *LGALS3* mRNA, which encodes galectin-3 (Gal-3), was increased 28-fold in high-grade astrocytoma compared to normal brain (Figure 4B; *P < .05). Moreover, relative protein expression of GAL-3, assessed via immunoblotting, was increased 100-fold in high-grade astrocytomas compared to normal brain and low-grade astrocytoma (Figure 4F; ***P < .001).

### The Canonical TGFB1 Pathway Was Upregulated in High-Grade Canine Astrocytoma

**Figure 5.**
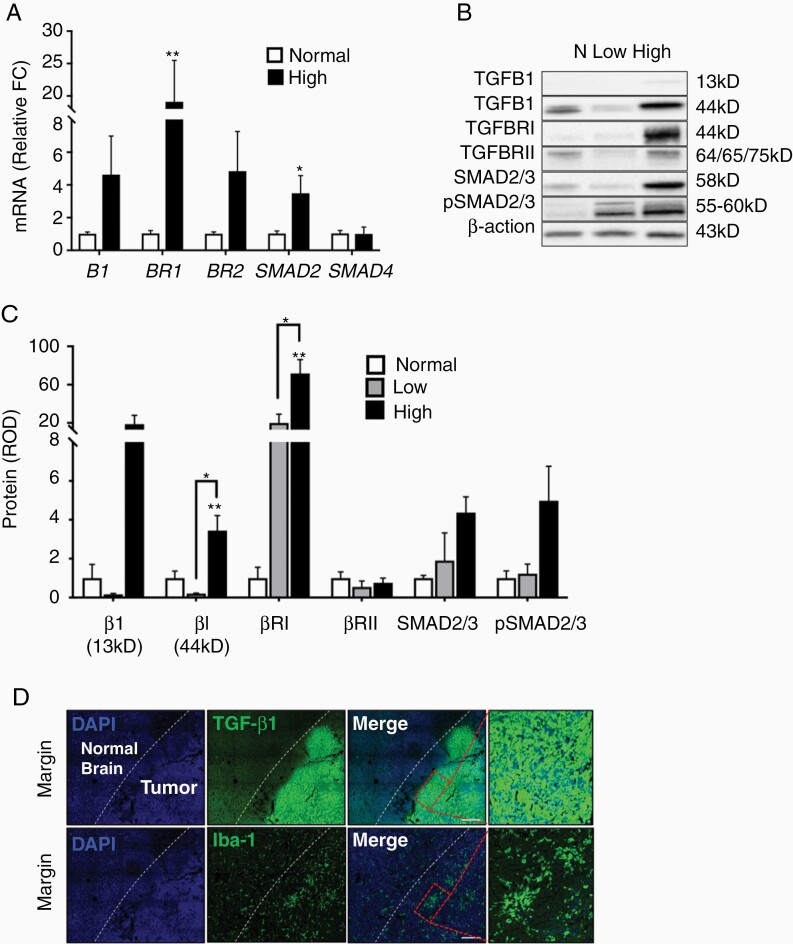
The canonical TGFB1 pathway was upregulated in high-grade canine astrocytoma. (A) mRNA levels of *BR1* and *SMAD2* were increased in high-grade astrocytoma tumor homogenate (*n* = 4) relative to normal cortex (*n* = 3). Comparisons based on unpaired *t*-test; *P < .05; **P < .01. (B) Representative immunoblots for proteins within the canonical TGFB1 pathway. (C) Relative optical density of mature TGFB1 (44 kD) and BR1 was increased in high-grade glioma tumor homogenates (*n* = 4) compared to normal cortex (*n* = 3) and low-grade astrocytoma (*n* = 3). Comparisons based on one-way analysis of variance with post hoc Tukey’s multiple comparisons test; *P < .05; **P < .01. Bars represent group mean with standard error of the mean. (D) Representative immunofluorescence of high-grade astrocytoma. The tumor margin is identified by the increased density of nuclei at the tumor edge (blue; DAPI). A robust increase in TGFB1 IR was observed close to the tumor margin, which correlated with robust Iba-1 IR on serial section, consistent with increased GAM density. Scale bars, 100 μm.

We quantified the expression of key molecules in the canonical TGFB1 signaling cascade. Similar to human glioma, mRNA levels of *TGFB1 receptor* (*BRI*; 17-fold; **P < .01) and the downstream transcription factor *SMAD2* (4-fold; *P < .05) were increased in high-grade astrocytoma compared to normal brain (Figure 5A). Relative protein expression of TGFBRI, as assessed via immunoblotting, was increased 60-fold in high-grade astrocytoma relative to normal brain (**P < .01) and low-grade glioma (*P < .05; Figure 5C). Moreover, the mature TGFB1 isoform (44 kD) was increased 3.5-fold in high-grade astrocytoma relative to normal brain (**P < .01) and low-grade glioma (*P < .05). Although protein expression of the cleaved, active form of TGFB1 (13 kD)^28^ was an average of 20-fold higher in high-grade astrocytoma, there was marked variability across tumor homogenates in this group.

To determine the spatial relationship between TGFB1 signaling and GAM density in high-grade astrocytoma, we performed immunofluorescence targeting Iba-1 and TGFB1. While weakly positive IR for TGFB1 was observed throughout the tumor mass, a marked increase in TGFB1 IR was noted at the leading edge of tumors (Figure 5D). Moreover, focal areas of increased GAM density were also noted adjacent to the leading edge of the tumor in high-grade astrocytomas (Figure 5D). These data suggest that TGFB1 expression is spatially heterogenous throughout the tumor and increased TGFB1 expression, at least partially, coincides with increased GAM density.

### TGFB1 Increases Canine Glioma Cell Migration and Invasion

**Figure 6.**
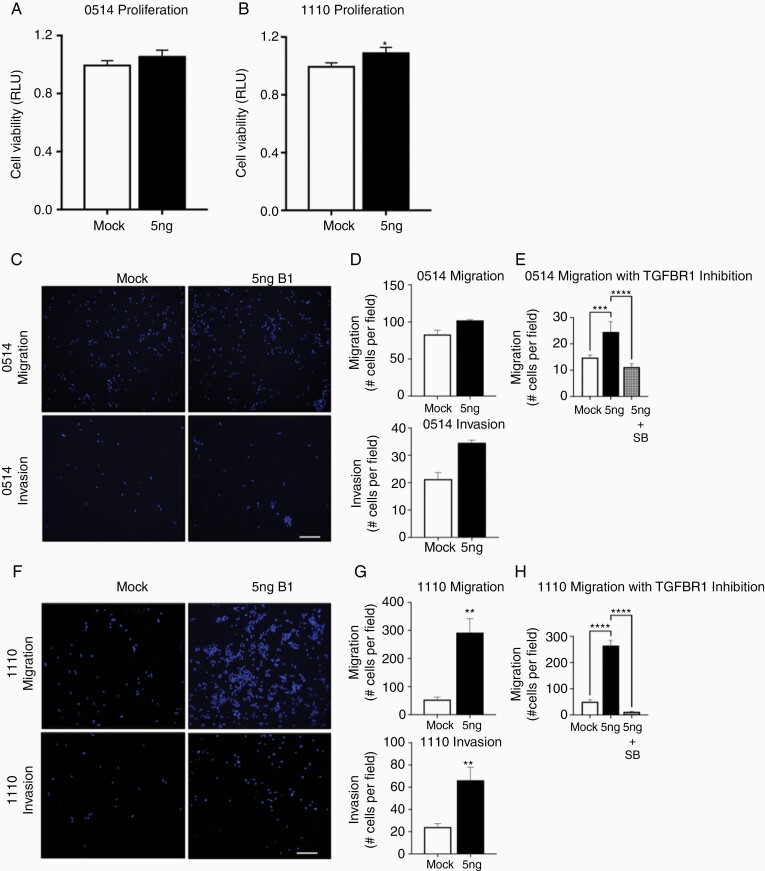
TGFB1 increases canine glioma stem cell migration and invasion. (A) Representative images of (A) 0514 cell viability was not affected by exposure to TGFB1 (5 ng/mL); (B) 1110 viability was modestly increased following exposure to TGFB1 (5 ng/mL); *P < .05. (C) 0514 and (F) 1110 migration and invasion following exposure to vehicle or TGFB1 (5 ng/mL). Cells were fixed in 4% paraformaldehyde and mounted with Vectashield with 4,5-diamidino-2phenylindole. Images were collected using a widefield fluorescent microscope (Model CTR 5000, Leica Microsystems, Inc.); scale bar 20 μm. Migration and invasion were increased in (D) 0514 and (G) 1110 cells following exposure to TGFB1 (5 ng/mL) *P < .05; **P < .01. (E) Pretreatment with 5 µM SB 431542, a TGFBR1 inhibitor, abolished the increased migration induced by TGFB1 in (E) 0514 and (F) 1110 canine astrocytoma cell lines; ***P < .001, ****P < .0001. Data represent one of 3 independent experiments.

Increased TGFB1 IR at the leading edge of the tumor mass led us to hypothesize that TGFB1 directly contributes to canine glioma invasion. Therefore, we assessed in vitro features of malignancy of 2 canine astrocytoma stem cell lines (0514, 1110) following exposure to TGFB1. TGFB1 exposure did not alter cellular viability of 0514 (1.0 ± 0 vs 1.06 ± 0; *P < .05; Figure 6A). However, a modest increase was observed in 1110 (1.11 ± 0 vs 1.0 ± 0; *P < .05; Figure 6B) cell viability following TGFB1 exposure. TGFB1 exhibited increased migration (102.0 ± 1.2 vs 83.0 ± 5.9; *P < .05) and invasion (34.5 ± 1.3 vs 21.3; *P < .05) in 0514 cells compared to control cells (Figure 6C and D), as well as increased migration (53.9 ± 11.2 vs 292 ± 60.0; **P < .01) and invasion (24.2 ± 2.2 vs 67.4 ± 8.5; *P < .05) in 1110 cells relative to control cells (Figure 6F and G). Importantly, the increased migration observed following TGFB1 treatment in both cell lines was abolished with TGFBR1 inhibitor pretreatment (0514: 14.8 ± 1 vs 24.5 ± 3.8 vs 11.2 ± 1.3; ***P < .001, ****P < .0001; 1110: 50.5 ± 8.2 vs 264.8 ± 19.7 vs 12.1 ± 1.8). Together, these data indicate that TGFB1 directly contributes to the malignant phenotype of canine astrocytoma cells.

## Discussion

In this study, we demonstrated that the GAM profile in high-grade canine astrocytoma is similar in several aspects to adult high-grade glioma. Increased GAM infiltration correlated with increases in several pro-tumorigenic molecules, including TGFB1, and a rich inflammatory cytokine milieu. Moreover, we observed that TGFB1 promotes tumor cell migration.

Comparable to human glioma, GAM density was increased 5- to 7-fold in high-grade canine astrocytoma. Surprisingly, GAM density was greatest in grade III, not grade IV, astrocytomas. This may suggest that the GAM response in canine glioma may not linearly correlate with tumor grade as in human glioma.^5^ The overlap in molecular signatures between microglia and macrophages often precludes the ability to distinguish between cell types in the tumor microenvironment. However, recent 2-photon brain imaging in rodent reporter models revealed that GAMs represent 2 morphologically distinct cell types—larger, branched microglia and smaller, minimally branched blood-derived macrophages.^29^ In the absence of lineage tracing and reporter models, we leveraged morphometric analysis to gain insight into the cellular origin of GAM. Several indices indicated that canine GAM had larger cell bodies with elongated projections compared to normal brain microglia. In particular, fiber length, while increased in all tumor grades, was nearly doubled in grade III astrocytoma. A similar GAM morphology was recently described in human glioma, whereby this appearance was determined to be consistent with microglia.^30^ Therefore, our data suggest that the predominant GAM population in grade III canine astrocytomas is microglia, not blood-derived macrophages. This finding is similar to recent rodent model data, suggesting that at least 65% of the GAM population is microglia.^31^ However, given the difference in average GAM morphological parameters between grade III and IV astrocytomas, it is tempting to speculate that grade IV astrocytomas may have increased infiltration of blood-derived macrophages, which would account for the reduction in the average cell area, fiber, and chord length in this population. Given the substantial differences in activation states between resident microglia and blood-derived macrophages,^31,32^ further quantitative evaluation of the ontogeny and the corresponding molecular profile of GAM is critical to guide the development of effective therapeutic strategies.

Increased microglial density has been observed in the contralateral normal-appearing cortical regions of rodent models^33^ and human patients.^34^ A positive relationship has been identified between the length of epilepsy history and microglia density in the cortex of human glioma patients.^34^ Where partial seizures unilaterally increased microglial density, generalized seizures increased microglia density bilaterally.^34^ We did not observe diffuse microglial activation in normal-appearing contralateral cortex in canine astrocytoma. However, mean microglial density in the normal-appearing contralateral cortex of grade III astrocytomas was elevated relative to normal brain (101.7 ± 49.83 vs 28.6 ± 16.2 cells/field). The overall increase was due to a single dog that had the greatest microglia density of all cases evaluated (180.1 cells/field). This dog had severe generalized seizures within 24 h of euthanasia and was the only dog with documented generalized seizure activity within 3 months prior to tissue collection in the morphometric cohort. Seizure-induced microglial activation has been well-described in dogs,^35,36^ thus it is plausible that secondary tumor effects (ie, seizures), but not the primary tumor, dictate the contralateral microglial response in canines and humans alike.

Our data demonstrate that several upregulated growth factors and cytokines within the tumor microenvironment are shared between canine and human astrocytomas. CCL2 is a key molecule mediating GAM recruitment in human glioma^37^ and expression is negatively associated with median survival time in human glioma patients.^38^ Similarly, CCL2 was highly expressed in high-grade canine astrocytoma and likely plays a critical role in GAM recruitment in canine tumors. Moreover, GAMs have been identified as a major *source* of CCL2 in glioblastoma multiforme.^39^ Rodent models have demonstrated that GAM-derived CCL2 is necessary for tumoral infiltration of T-regulatory cells.^39^ Therefore, it is plausible that canine GAMs are also a source of CCL2 within canine astrocytomas, similar to human astrocytomas. Given the recent observation of the abundance of CD4+ cells in canine astrocytoma,^21^ further evaluation of the source and specific role(s) of CCL2 in canine astrocytoma is warranted.

The complexity of the human glioma molecular profile is mirrored in canine astrocytoma, with upregulation of several molecules that promote the malignant astrocytoma phenotype. Similar to human glioma,^40^ HGFR, a high-affinity tyrosine kinase receptor for HGF, was the most abundantly increased molecule evaluated in high-grade canine astrocytoma (Figure 4). Despite variability in HGF abundance, the incredible magnitude of HGFR expression suggests that the HGF/HGFR signaling axis is relevant in canine astrocytoma pathogenesis. Tumor-derived HGF has been shown to facilitate microglia migration^41^ through paracrine signaling, as well as promote glioma invasion and chemoresistance through autocrine signaling.^40,42^ It is also possible that microglia are a source of HGF in the tumor environment, as TGFB isoforms induce microglia production of HGF.^43^ Importantly, therapeutic targeting of HGF or HGFR through antibodies or small-molecule inhibitors has shown efficacy in numerous preclinical studies, but the therapeutic benefit has failed to translate to human patients. Given our findings, naturally occurring canine astrocytomas may provide a platform for further preclinical therapeutic targeting of this signaling axis.

As in human glioma,^44^ several molecules of the IL-1 cytokine superfamily were highly expressed in high-grade canine astrocytoma. In addition to the direct promotion of glioma growth and invasion,^45,46^ IL-1 cytokines are necessary for VEGF production and angiogenesis in glioma.^47^ Indeed, VEGF expression was highly abundant in our sample cohort. Thus, these data suggest that the IL-1 signaling axis is quite relevant in canine astrocytoma and contributes to the interdependent, pro-tumorigenic signaling milieu. Moreover, while these molecules may be derived from multiple sources in the glioma microenvironment, microglia are likely key players in establishing the pro-tumorigenic molecular profile of canine astrocytoma.

TGFB1 is an abundant, pro-tumorigenic cytokine in human glioma.^48^ Here we demonstrate a striking upregulation of the canonical TGFB signaling axis in canine high-grade astrocytoma. However, TGFB1 IR was heterogeneously distributed throughout high-grade astrocytomas. We observed a marked upregulation of IR at the leading edge of the tumors, which corresponded to a focal area of increased microglia density (Figure 5). Human tumor-derived TGFB1 stimulates GAM to produce molecules promoting glioma growth^9^ and invasion.^10^ Additionally, GAMs are also a *source* of TGFB1, serving to promote glioma cell invasion.^10,48^ While this study does not ascertain the source of TGFB1 in canine astrocytomas, we have demonstrated that canine astrocytoma cells increase migration, invasion, and to a lesser extent, proliferation, following exposure to TGFB1, consistent with human data.^10^ Interestingly, there was a negative correlation between the tumoral abundance of active TGFB1 (13 kD) and survival time in dogs with high-grade astrocytoma (*r*_s_: −1.0’ *P* = .08; *n* = 4; Supplementary Figure 2), just as in human glioma patients.^49^ Survival time in veterinary medicine is dictated by the owner’s wishes for euthanasia and is not a good measure of disease end-stage in all cases. However, within the 4 cases presented here, euthanasia was elected at the time of unmanageable clinical signs and may more closely represent progression-free interval. Either way, this relationship highlights the potential importance of TGFB signaling on the malignant phenotype in canine astrocytoma and further underscores the similarities between canine astrocytoma and human glioma.

These data provide further evidence that naturally occurring canine astrocytomas are a relevant translational model for human glioma. Both human and canine high-grade astrocytomas are characterized by an increased density of enlarged and elongated GAM and an abundance of several pro-tumorigenic molecules, including the highly active TGFB signaling axis which directly promotes the malignant astrocytoma phenotype. Canine spontaneous astrocytomas appear to have the core common features supporting their validity for the assessment of immunomodulatory therapeutic approaches targeting GAM and the TGFB axis. Therefore, naturally occurring, heterogenous canine astrocytomas, with shared environmental influence on humans, may provide a more accurate large animal model platform for the development of therapeutic approaches for human patients.

## Supplementary Material

vdab062_suppl_Supplementary_Material

## Notes

**Authorship Statement.** All authors have significantly contributed to this manuscript. Experimental design: R.T., A.C.G. and C.M.T. Implementation: R.T., A.C.G., P.D., K.W., N.V., B.S., C.F.L., O.N., B.C., K.V., M.K., V.M., P.J.L. and C.M.T. Data analysis and interpretation: R.T., A.C.G., J.S., P.J.L. and C.M.T. Manuscript writing: R. T., A.C.G., and C.M.T. Final approval of manuscript: all authors.

This work has been partially presented in abstract form at the 2019 ACVIM Forum, Phoenix, AZ.

## References

[1] Stupp R , Taillibert S, Kanner A, et al. Effect of tumor-treating fields plus maintenance temozolomide vs maintenance temozolomide alone on survival in patients with glioblastoma: a randomized clinical trial. JAMA. 2017;318(23):2306–2316. 29260225 10.1001/jama.2017.18718 PMC5820703

[2] Cristescu R , Mogg R, Ayers M, et al. Pan-tumor genomic biomarkers for PD-1 checkpoint blockade-based immunotherapy. Science. 2018;362(6411):eaar3593. 30309915 10.1126/science.aar3593 PMC6718162

[3] Gutmann DH , McLellan MD, Hussain I, et al. Somatic neurofibromatosis type 1 (NF1) inactivation characterizes NF1-associated pilocytic astrocytoma. Genome Res. 2013;23(3):431–439. 23222849 10.1101/gr.142604.112 PMC3589532

[4] Wu A , Wei J, Kong LY, et al. Glioma cancer stem cells induce immunosuppressive macrophages/microglia. Neuro Oncol. 2010;12(11):1113–1125. 20667896 10.1093/neuonc/noq082 PMC3098021

[5] Sørensen MD , Dahlrot RH, Boldt HB, Hansen S, Kristensen BW. Tumour-associated microglia/macrophages predict poor prognosis in high-grade gliomas and correlate with an aggressive tumour subtype. Neuropathol Appl Neurobiol. 2018;44(2):185–206. 28767130 10.1111/nan.12428

[6] Markovic DS , Glass R, Synowitz M, Rooijen Nv, Kettenmann H. Microglia stimulate the invasiveness of glioma cells by increasing the activity of metalloprotease-2. J Neuropathol Exp Neurol. 2005;64(9):754–762. 16141784 10.1097/01.jnen.0000178445.33972.a9

[7] Li W , Graeber MB. The molecular profile of microglia under the influence of glioma. Neuro Oncol. 2012;14(8):958–978. 22573310 10.1093/neuonc/nos116 PMC3408253

[8] Fadok VA , Bratton DL, Konowal A, Freed PW, Westcott JY, Henson PM. Macrophages that have ingested apoptotic cells in vitro inhibit proinflammatory cytokine production through autocrine/paracrine mechanisms involving TGF-beta, PGE2, and PAF. J Clin Invest. 1998;101(4):890–898. 9466984 10.1172/JCI1112 PMC508637

[9] Brandenburg S , Müller A, Turkowski K, et al. Resident microglia rather than peripheral macrophages promote vascularization in brain tumors and are source of alternative pro-angiogenic factors. Acta Neuropathol. 2016;131(3):365–378. 26718201 10.1007/s00401-015-1529-6

[10] Wesolowska A , Kwiatkowska A, Slomnicki L, et al. Microglia-derived TGF-beta as an important regulator of glioblastoma invasion—an inhibition of TGF-beta-dependent effects by shRNA against human TGF-beta type II receptor. Oncogene. 2008;27(7):918–930. 17684491 10.1038/sj.onc.1210683

[11] Han J , Alvarez-Breckenridge CA, Wang QE, Yu J. TGF-β signaling and its targeting for glioma treatment. Am J Cancer Res. 2015;5(3):945–955. 26045979 PMC4449428

[12] Butowski N , Colman H, De Groot JF, et al. Orally administered colony stimulating factor 1 receptor inhibitor PLX3397 in recurrent glioblastoma: an Ivy foundation early phase clinical trials consortium phase II study. Neuro Oncol. 2016;18(4):557–564. 26449250 10.1093/neuonc/nov245 PMC4799682

[13] Datto MB , Li Y, Panus JF, Howe DJ, Xiong Y, Wang XF. Transforming growth factor beta induces the cyclin-dependent kinase inhibitor p21 through a p53-independent mechanism. Proc Natl Acad Sci U S A. 1995;92(12):5545–5549. 7777546 10.1073/pnas.92.12.5545 PMC41732

[14] Dumont N , Bakin AV, Arteaga CL. Autocrine transforming growth factor-beta signaling mediates Smad-independent motility in human cancer cells. J Biol Chem. 2003;278(5):3275–3285. 12421823 10.1074/jbc.M204623200

[15] Dolecek TA , Propp JM, Stroup NE, Kruchko C. CBTRUS statistical report: primary brain and central nervous system tumors diagnosed in the United States in 2005–2009. Neuro Oncol. 2012;14 (suppl 5):v1–49. 23095881 10.1093/neuonc/nos218 PMC3480240

[16] Song RB , Vite CH, Bradley CW, Cross JR. Postmortem evaluation of 435 cases of intracranial neoplasia in dogs and relationship of neoplasm with breed, age, and body weight. J Vet Intern Med. 2013;27(5):1143–1152. 23865437 10.1111/jvim.12136

[17] Mariani CL , Schubert TA, House RA, et al. Frameless stereotactic radiosurgery for the treatment of primary intracranial tumours in dogs. Vet Comp Oncol. 2015;13(4):409–423. 24007303 10.1111/vco.12056

[18] Bentley RT . Magnetic resonance imaging diagnosis of brain tumors in dogs. Vet J. 2015;205(2):204–216. 25792181 10.1016/j.tvjl.2015.01.025

[19] Koehler JW , Miller AD, Miller CR, et al. A revised diagnostic classification of canine glioma: towards validation of the canine glioma patient as a naturally occurring preclinical model for human glioma. J Neuropathol Exp Neurol. 2018;77(11):1039–1054. 30239918 10.1093/jnen/nly085 PMC6181180

[20] Boudreau CE , York D, Higgins RJ, LeCouteur RA, Dickinson PJ. Molecular signalling pathways in canine gliomas. Vet Comp Oncol. 2017;15(1):133–150. 25808605 10.1111/vco.12147

[21] Amin SB , Anderson KJ, Boudreau CE, et al. Comparative molecular life history of spontaneous canine and human gliomas. Cancer Cell. 2020;37(2):243–257.e247. 32049048 10.1016/j.ccell.2020.01.004 PMC7132629

[22] Louis DN , Perry A, Reifenberger G, et al. The 2016 World Health Organization classification of tumors of the central nervous system: a summary. Acta Neuropathol. 2016;131(6):803–820. 27157931 10.1007/s00401-016-1545-1

[23] Livak K. Comparative Ct Method. ABI Prism 7700 Sequence Detection System. User Bulletin no. 2 PE Applied Biosystems, CA, USA. 1997.

[24] Toedebusch RG , Ruegsegger GN, Braselton JF, et al. AMPK agonist AICAR delays the initial decline in lifetime-apex V̇o2 peak, while voluntary wheel running fails to delay its initial decline in female rats. Physiol Genomics. 2016;48(2):101–115. 26578698 10.1152/physiolgenomics.00078.2015

[25] Lum-Naihe K , Toedebusch R, Mahmood A, et al. Cardiovascular disease progression in female Zucker Diabetic Fatty rats occurs via unique mechanisms compared to males. Sci Rep. 2017;7(1):17823. 29259233 10.1038/s41598-017-18003-8 PMC5736602

[26] Li Y , Sun T, Chen Z, Shao Y, Huang Y, Zhou Y. Characterization of a new human astrocytoma cell line SHG140: cell proliferation, cell phenotype, karyotype, STR markers and tumorigenicity analysis. J Cancer. 2021;12(2):371–378. 33391433 10.7150/jca.40802 PMC7738992

[27] Toedebusch CM , Garcia VB, Snyder JC, et al. Lumbar spinal cord microglia exhibited increased activation in aging dogs compared with young adult dogs. Geroscience. 2020;42(1):169–182. 31828496 10.1007/s11357-019-00133-8 PMC7031472

[28] Garris CS , Arlauckas SP, Kohler RH, et al. Successful anti-PD-1 cancer immunotherapy requires T cell-dendritic cell crosstalk involving the cytokines IFN-γ and IL-12. Immunity. 2018;49(6):1148–1161.e1147. 30552023 10.1016/j.immuni.2018.09.024 PMC6301092

[29] Chen Z , Ross JL, Hambardzumyan D. Intravital 2-photon imaging reveals distinct morphology and infiltrative properties of glioblastoma-associated macrophages. Proc Natl Acad Sci U S A. 2019;116(28):14254–14259. 31235603 10.1073/pnas.1902366116 PMC6628659

[30] Saavedra-López E , Roig-Martínez M, Cribaro GP, et al. Phagocytic glioblastoma-associated microglia and macrophages populate invading pseudopalisades. Brain Commun. 2020;2(1):fcz043. 32954312 10.1093/braincomms/fcz043 PMC7491442

[31] Bowman RL , Klemm F, Akkari L, et al. Macrophage ontogeny underlies differences in tumor-specific education in brain malignancies. Cell Rep. 2016;17(9):2445–2459. 27840052 10.1016/j.celrep.2016.10.052 PMC5450644

[32] Müller S , Kohanbash G, Liu SJ, et al. Single-cell profiling of human gliomas reveals macrophage ontogeny as a basis for regional differences in macrophage activation in the tumor microenvironment. Genome Biol. 2017;18(1):234. 29262845 10.1186/s13059-017-1362-4 PMC5738907

[33] Crommentuijn MHW , Schetters STT, Dusoswa SA, Kruijssen LJW, Garcia-Vallejo JJ, van Kooyk Y. Immune involvement of the contralateral hemisphere in a glioblastoma mouse model. J Immunother Cancer. 2020;8(1):e000323. 32303613 10.1136/jitc-2019-000323 PMC7204813

[34] Su Z , Frederico R, Hinz R, et al. Microglial activation in normal-appearing brain regions of patients with cerebral glioma: a cross-sectional study Lancet. 2017;389(S92).

[35] Sakurai M , Morita T, Takeuchi T, Shimada A. Relationship of angiogenesis and microglial activation to seizure-induced neuronal death in the cerebral cortex of Shetland Sheepdogs with familial epilepsy. Am J Vet Res. 2013;74(5):763–770. 23627390 10.2460/ajvr.74.5.763

[36] Stein VM , Genini S, Puff C, Baumgärtner W, Tipold A. Seizure activity in dogs is associated with enhanced TIMP-2 expression of microglia. Vet Immunol Immunopathol. 2012;146(2):101–105. 22381031 10.1016/j.vetimm.2012.02.003

[37] Platten M , Kretz A, Naumann U, et al. Monocyte chemoattractant protein-1 increases microglial infiltration and aggressiveness of gliomas. Ann Neurol. 2003;54(3):388–392. 12953273 10.1002/ana.10679

[38] Chen Z , Feng X, Herting CJ, et al. Cellular and molecular identity of tumor-associated macrophages in glioblastoma. Cancer Res. 2017;77(9):2266–2278. 28235764 10.1158/0008-5472.CAN-16-2310 PMC5741820

[39] Chang AL , Miska J, Wainwright DA, et al. CCL2 produced by the glioma microenvironment is essential for the recruitment of regulatory t cells and myeloid-derived suppressor cells. Cancer Res. 2016;76(19):5671–5682. 27530322 10.1158/0008-5472.CAN-16-0144 PMC5050119

[40] Kim KH , Seol HJ, Kim EH, et al. Wnt/β-catenin signaling is a key downstream mediator of MET signaling in glioblastoma stem cells. Neuro Oncol. 2013;15(2):161–171. 23258844 10.1093/neuonc/nos299 PMC3548587

[41] Badie B , Schartner J, Klaver J, Vorpahl J. In vitro modulation of microglia motility by glioma cells is mediated by hepatocyte growth factor/scatter factor. Neurosurgery. 1999;44(5):1077–1082; discussion 1082-1073. 10232541 10.1097/00006123-199905000-00075

[42] Dong F , Eibach M, Bartsch JW, et al. The metalloprotease-disintegrin ADAM8 contributes to temozolomide chemoresistance and enhanced invasiveness of human glioblastoma cells. Neuro Oncol. 2015;17(11):1474–1485. 25825051 10.1093/neuonc/nov042 PMC4648299

[43] Lalive PH , Paglinawan R, Biollaz G, et al. TGF-beta-treated microglia induce oligodendrocyte precursor cell chemotaxis through the HGF-c-Met pathway. Eur J Immunol. 2005;35(3):727–737. 15724248 10.1002/eji.200425430

[44] Sasaki A , Tamura M, Hasegawa M, Ishiuchi S, Hirato J, Nakazato Y. Expression of interleukin-1beta mRNA and protein in human gliomas assessed by RT-PCR and immunohistochemistry. J Neuropathol Exp Neurol. 1998;57(7):653–663. 9690669 10.1097/00005072-199807000-00002

[45] Oelmann E , Kraemer A, Serve H, et al. Autocrine interleukin-1 receptor antagonist can support malignant growth of glioblastoma by blocking growth-inhibiting autocrine loop of interleukin-1. Int J Cancer. 1997;71(6):1066–1076. 9185713 10.1002/(sici)1097-0215(19970611)71:6<1066::aid-ijc25>3.0.co;2-a

[46] Fathima Hurmath K , Ramaswamy P, Nandakumar DN. IL-1β microenvironment promotes proliferation, migration, and invasion of human glioma cells. Cell Biol Int. 2014;38(12):1415–1422. 25053165 10.1002/cbin.10353

[47] Voronov E , Shouval DS, Krelin Y, et al. IL-1 is required for tumor invasiveness and angiogenesis. Proc Natl Acad Sci U S A. 2003;100(5):2645–2650. 12598651 10.1073/pnas.0437939100 PMC151394

[48] Ye XZ , Xu SL, Xin YH, et al. Tumor-associated microglia/macrophages enhance the invasion of glioma stem-like cells via TGF-β1 signaling pathway. J Immunol. 2012;189(1):444–453. 22664874 10.4049/jimmunol.1103248

[49] Roy LO , Poirier MB, Fortin D. Differential expression and clinical significance of transforming growth factor-beta isoforms in GBM tumors. Int J Mol Sci. 2018;19(4):1113. 10.3390/ijms19041113 PMC5979513 29642484

