# Supplementary material for: Glioma-associated microglia/macrophages augment tumorigenicity in canine astrocytoma, a naturally occurring model of human glioma
Source: Neurooncol Adv. 2021 May 4;3(1):vdab062. doi: 10.1093/noajnl/vdab062 (PMC8193901; doi:10.1093/noajnl/vdab062)
Supplement: vdab062_suppl_Supplementary_Material [file vdab062_suppl_supplementary_material.zip › vdab062_suppl_Supplementary_Materials_S2.docx]

Supplementary Table 1: Case information for each study cohort.

Supplementary Table 2: Primer sets used for realtime PCR reactions. Accession numbers are provided for each gene of interest. Primer sets were generated using NCBI’s primer design tool. All primer sets were validated as described in Methods.

Supplementary Table 3: Cytokines analyzed via RayBiotech, Inc. and associated references in manuscript.
