# Supplementary material for: Glioma-associated microglia/macrophages augment tumorigenicity in canine astrocytoma, a naturally occurring model of human glioma
Source: Neurooncol Adv. 2021 May 4;3(1):vdab062. doi: 10.1093/noajnl/vdab062 (PMC8193901; doi:10.1093/noajnl/vdab062)
Supplement: vdab062_suppl_Supplementary_Material [file vdab062_suppl_supplementary_material.zip › vdab062_suppl_Supplementary_Materials_S1.docx]

Supplementary Methods

Immunofluorescence

Previously formalin fixed paraffin embedded tissue was obtained from the VMTH pathology service. Tissue was sectioned to 10 µm and mounted to poly-L-lysine treated microscope slides. The tissue was deparaffinized using xylene and decreasing concentrations of ethanol. Antigen retrieval was performed using Dako Antigen Retrieval solution (DAKO) at 95°C for 20 minutes. tissue was permeabilized with Tris-buffered saline with 0.1% Triton X-100 (TBST). Tissue was blocked using Normal Goat serum (5%), bovine serum albumin (1%) in TBST overnight at 4°C. The following primary antibodies were used: rabbit polyclonal anti-ionized calcium-binding adaptor molecule (Iba-1; microglia/macrophage) (Wako Pure Chemical Industries, Ltd., Chuo-Ku, Osaka, Japan; 019-19741, 1:1000) and mouse monoclonal anti-glial fibrillary acidic protein (GFAP; astrocytes) (Encor Biotechnology, Gainseville, FL; MCA-5C10, 1:1000). Tissue was incubated in primary antibody solution in fresh blocking buffer, or blocking buffer alone for negative controls, overnight at 4°C. The sections were washed three times for 5 minutes in TBST at room temperature, and incubated with IgG (heavy and light) anti-mouse, anti-chicken or anti-rabbit secondary antibodies that were conjugated with either Alexa Fluor 488 or 555 (Molecular Probes, Invitrogren, Carlsbad, CA, USA; 1:1000) diluted in fresh blocking buffer at RT for 1 hour. Tissue was then washed three times for 5 minutes at room temperature. Tissue was mounted with Vectashield with 4’,5-diamidino-2phenylindole (DAPI) (Vector Labs, Burlingame, CA, USA).

qRT-PCR

Total RNA was isolated using the Direct-zol MiniPrep kit (Zymo Research, Irvine, CA, USA) according to manufacturer’s specifications, and DNase treatment was carried out on the column before RNA elution. Using one microgram purified RNA, cDNA was reverse transcribed using the High-Capacity cDNA Reverse Transcription Kit (Thermo Fisher – Applied Biosystems, Waltham, MA, USA). Primers were purchased from Integrated DNA Technologies (Coralville, Iowa, USA) and validated using melt curves and gel electrophoresis. Validation for each set of primers was performed using a 4x cDNA serial dilution series from control dog brain as a template. We evaluated the efficiency and fit of the curves generated; primer sets that did not produce an efficiency of at least 0.9 and an R^2^ value of 0.95 from the cDNA dilution series were rejected. Only experimental quantification cycle (Cq) values that fell within the boundaries of the validated curves were used for analysis.

qPCR reactions consisted of primer pairs at a final concentration of 200nM, 50ng cDNA template, and SSoAdvanced Universal SYBR Green Supermix (Bio-Rad, Hercules, CA, USA) per manufacturer’s protocol. Reactions were carried out on a CFXConnect (Bio-Rad) machine with a three-step cycle of 95ºC-15s, 60ºC-20s, 72ºC-20s, followed by a melt curve ramp from 65ºC to 95ºC. Data were acquired during the 72ºC step, and every 0.5ºC of the melt curve. All reactions were run as 20μl triplicates, and the average Cq used as the data point for a given sample. Care was taken at each step to minimize assay variability: samples were processed in parallel, the same batch of reverse transcriptase was used for all samples, and PCR runs were designed to maximize the number of samples run in each batch. mRNA expression values were quantified by the 2^-ΔΔCt^ method, whereby ΔCT = 18S Ct – gene of interest Ct.

Western Blot Analysis

Protein extracts from tumor samples were obtained via homogenization of 30 mg tissue on ice in 300 μL RIPA buffer containing 1X Halt Protease Inhibitor Cocktail (Thermo Fisher Scientific, Waltham, MA, USA) and 1X Halt Phosphatase inhibitor cocktails (Thermo Fisher Scientific, USA). Protein concentration was determined using the BCA assay (Thermo Fisher Scientific, USA). Proteins (60 μg) were separated on 10% SDS-polyacrylamide gels followed, and transferred to nitrocellulose membranes. Membranes were washed three times in TBS with 1% Tween^20^ (TBS-Tween^20^) at room temperature and blocked in blocking buffer (5% fat free milk diluted in TBS-Tween^20^) for 1 hour at room temperature. Membranes were then incubated in the following primary antibody solutions diluted in fresh blocking buffer at 4 °C overnight: TGF-𝛽1 (Abcam, Cambridge, MA, USA; ab92486, 1:2500), TGFBR1 (Santa Cruz Biotechnology, Inc. Dallas, TX, USA; sc-101574, 1:200), TGFBR2 (Santa Cruz Biotechnology; sc-17791, 1:100), SMAD2/3 (BD Biosciences, San Jose, CA. USA; 610842, 1:1000), and phospho-SMAD2/3 (Millipore, Burlington, MA, USA; 04-953, 1:500). Membranes were then washed three times in TBS-Tween^20^, and incubated with species-specific HRP-linked secondary antibodies (Jackson [Immuno](https://www.sciencedirect.com/topics/neuroscience/intravenous-immunoglobulin) Research Labs Inc., PA, USA; 1:20,000) in 1% BSA at RT for 2 h.  Chemiluminescence images were acquired using ChemiDoc XRS+ System (Bio-Rad, USA) after ECL reagent incubation (Thermo Fisher Scientific, USA). Images were analyzed using ImageLab software (Bio-Rad, USA). Data were expressed as relative [optical densities](https://www.sciencedirect.com/topics/biochemistry-genetics-and-molecular-biology/optical-density) normalized to control dog tissue.

Canine astrocytoma cell lines

Primary canine astrocytoma cell lines were cultured under the following serum free conditions, Neurobasal-A media (Thermo Fisher Scientific; 10888022), B27 supplement (Thermo Fisher Scientific; 12587010), N2 supplement (Thermo Fisher Scientific; 17502048), and 0.5X L-glutamine (Thermo Fisher Scientific; 25030081). The following growth factors were supplemented; 25 ng/ml bFGF (R&D Systems, Inc., Minneapolis, MN, USA; 233-FB-025), 25 ng/ml EGF (R&D Systems, Inc., USA; 236-EG-200), and PDGF (Gemini Bio Products, West Sacramento, CA, USA; 300-178P).

*Cell Viability*

Cell viability was performed using the Cell Titer Glo® 2.0 Assay (Promega) according to the manufacturers protocol. Briefly, 25,000 cells canine astrocytoma cells (GSC0514 and GSC1110) were seeded per well of 96-well black-sided plates. Cells were treated with 5ng/mL human recombinant TGFB1 (PeproTech, Inc, Cranbury, NJ, USA; 100-21) or PBS (mock; Thermo Fisher Scientific 14190-144) once every 24 hours for a total of 2 treatments (48 hour total incubation). Titer Glo® reagent was added to each well, the plate was incubated for 10 minutes at RT on a plate shaker, followed by luminescence recording via plate reader (BioTek 800TS). Optical densities were recorded for 6 replicates per condition and three independent experiments were performed.

*Transwell migration and invasion assay*

Canine astrocytoma cells were treated with 5ng/mL human recombinant TGFB1 (PeproTech, Inc, Cranbury, NJ, USA; 100-21) or PBS (mock) as described for cell viability analysis. Additionally, these experiments were repeated with 1hr. pretreatment with a potent and selective TGFBR1 inhibitor (5 µM SB 431542; Tocris, Bio-Techne Corporation, Minneapolis, MN, USA). Cells (100,000 per well) were suspended in serum-free culture medium and seeded into either migration or invasion transwell inserts (pore size 8 µm). Invasion assays contained Matrigel coating suspended above the pores. After 24 hours, inserts were removed, the top of each insert was swabbed to remove non-migrated cells, and the remaining cells attached to the bottom of the insert were fixed using 4% paraformaldehyde. Membranes were excised from inserts and mounted onto microscope slides using mounting medium containing DAPI (4′,6-diamidino-2-phenylindole). Nine photographs were taken per membrane, with three technical replicates per experiment, using a brightfield microscope (Leica, DM5000 B). Cells were recognized by positive DAPI IR and quantified via ImageJ custom macro.
