# Supplementary figures and images for: Glioma-associated microglia/macrophages augment tumorigenicity in canine astrocytoma, a naturally occurring model of human glioma
Source: Neurooncol Adv. 2021 May 4;3(1):vdab062. doi: 10.1093/noajnl/vdab062 (PMC8193901; doi:10.1093/noajnl/vdab062)

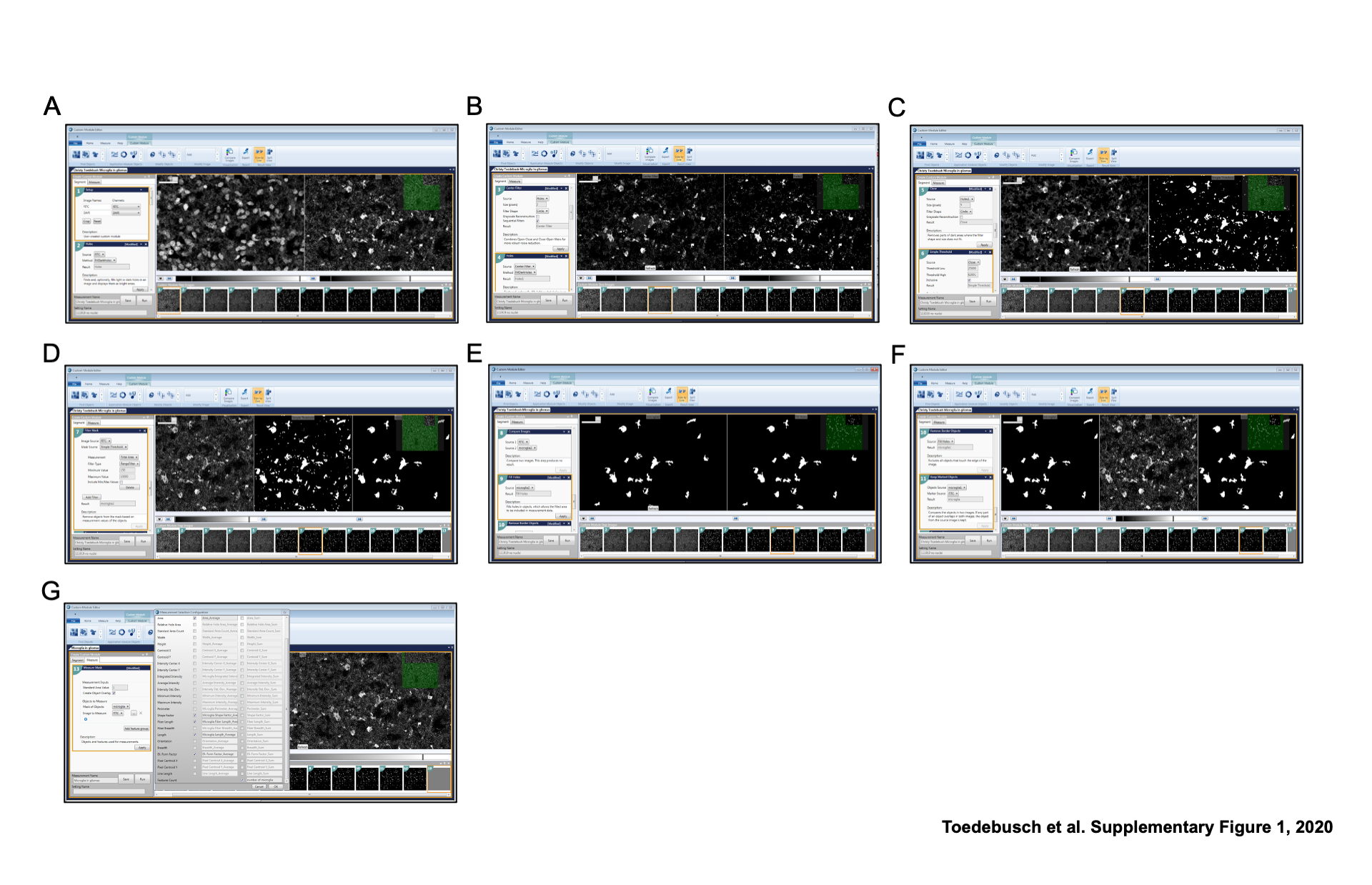

Supplement: vdab062_suppl_Supplementary_Material [file vdab062_suppl_supplementary_material.zip › vdab062_suppl_Supplementary_Figure_S1.tiff]

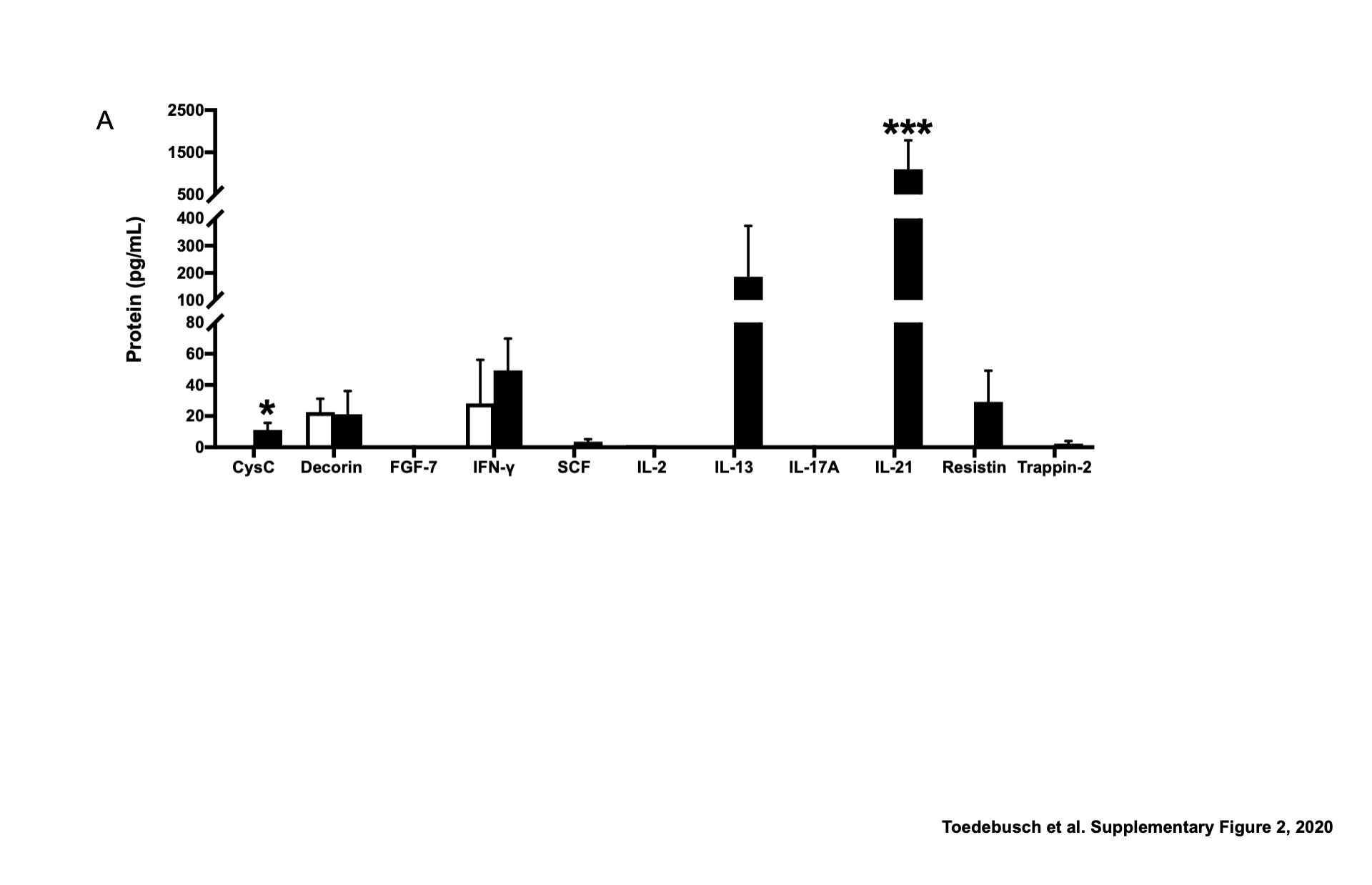

Supplement: vdab062_suppl_Supplementary_Material [file vdab062_suppl_supplementary_material.zip › vdab062_suppl_Supplementary_Figure_S2.tiff]

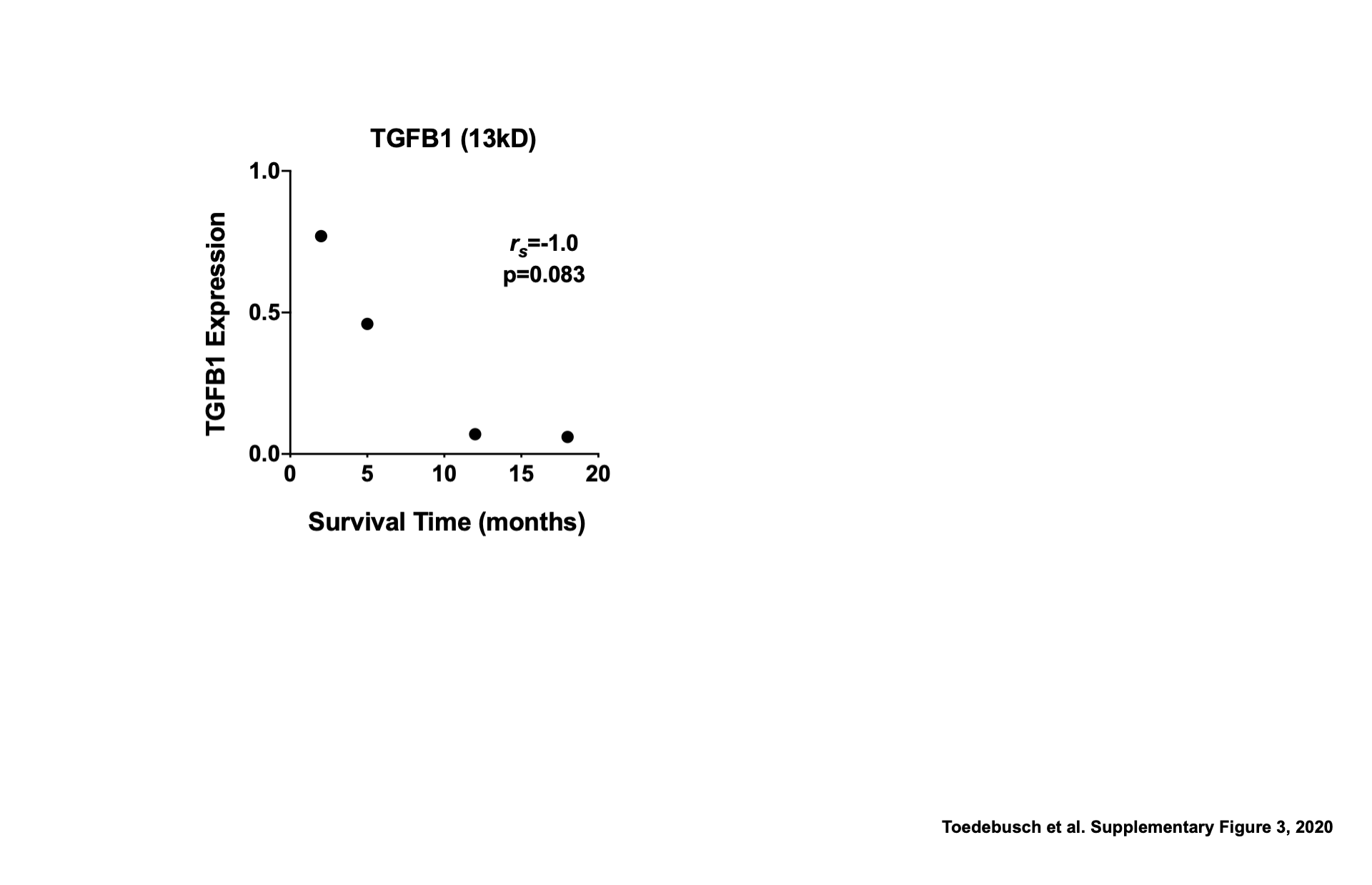

Supplement: vdab062_suppl_Supplementary_Material [file vdab062_suppl_supplementary_material.zip › vdab062_suppl_Supplementary_Figure_S3.tiff]
